# Clustering-based identification of clonally-related immunoglobulin gene sequence sets

**DOI:** 10.1186/1745-7580-6-S1-S4

**Published:** 2010-09-27

**Authors:** Zhiliang Chen, Andrew M Collins, Yan Wang, Bruno A Gaëta

**Affiliations:** 1 School of Computer Science and Engineering, University of New South Wales, NSW 2052, Australia; 2 School of Biotechnology and Biomolecular Sciences, University of New South Wales, NSW 2052, Australia

## Abstract

**Background:**

Clonal expansion of B lymphocytes coupled with somatic mutation and antigen selection allow the mammalian humoral immune system to generate highly specific immunoglobulins (IG) or antibodies against invading bacteria, viruses and toxins. The availability of high-throughput DNA sequencing methods is providing new avenues for studying this clonal expansion and identifying the factors guiding the generation of antibodies. The identification of groups of rearranged immunoglobulin gene sequences descended from the same rearrangement (clonally-related sets) in very large sets of sequences is facilitated by the availability of immunoglobulin gene sequence alignment and partitioning software that can accurately predict component germline gene, but has required painstaking visual inspection and analysis of sequences.

**Results:**

We have developed and implemented an algorithm for identifying sets of clonally-related sequences in large human immunoglobulin heavy chain gene variable region sequence sets. The program processes sequences that have been partitioned using iHMMune-align, and uses pairwise comparisons of CDR3 sequences and similarity in IGHV and IGHJ germline gene assignments to construct a distance matrix. Agglomerative hierarchical clustering is then used to identify likely groups of clonally-related sequences.  The program is available for download from http://www.cse.unsw.edu.au/~ihmmune/ClonalRelate/ClonalRelate.zip.

**Conclusions:**

The method was evaluated on several benchmark datasets and provided a more accurate and considerably faster identification of clonally-related immunoglobulin gene sequences than visual inspection by domain experts.

## Background

The human immune system has the ability to produce millions of different types of antibodies in the defence against bacteria, virus and toxins. Immunoglobulin heavy and light chain gene rearrangement happens during the early differentiation of the B cell precursors.  The rearranged immunoglobulin heavy (IGH) chain is formed by recombination of genes selected from three sets of germline genes: variable (immunoglobulin heavy chain variable, IGHV), diversity (IGHD) and joining (IGHJ) [1]. Additional diversity is introduced by N nucleotide addition (the process of adding non-germline-encoded nucleotides at the time of gene rearrangement) and, during clonal selection, by the introduction of point mutations through the process of somatic hypermutation. The accumulation of mutations during clonal expansion improves antigen binding affinity and results in the formation of clonally-related immunoglobulin gene sets, each derived from a single germline rearrangement.

The development of ultra-deep DNA sequencing technologies is opening a powerful new avenue of investigation into the B cell-mediated immune response, by enabling the characterisation of antibody diversity in individuals [2].The identification of sets of clonally-related sequences is a significant component of this analysis as it allows determining the shape of the clonal expansion in response to antigen exposure and other conditions [3]. This information may have a critical bearing on the clinical significance imputed to clonal B cells in the blood, in terms of their ability to persist and potentially mediate relapse of disease, or in auto-immune diseases [4,5].

Previous studies have demonstrated the importance and potential of accurate alignment and analysis for studying the immune response, using software such as IMGT/V-QUEST [6] , SoDA [7], iHMMune-align [8], Ab-origin [9], etc. However, none of these programs allow the direct identification of clonally-related immunoglobulin gene sets.

The third complementarity determining region (CDR3) is a highly variable region in V domain. This region encodes a protein loop that lies at the centre of the antigen binding site [10,11], and its length and composition influence antigen binding [12]. The CDR3 of an IGH variable domain (VH) spans the VH- DH- JH joint, with interposed N region addition, and is the most variable region of the heavy chain genes. As such it has the greatest potential for the identification of clonal relationships between sequences. Previous studies [13,14] have demonstrated that antigen receptor gene arrangement and B cell diversification can be analyzed by modelling the length distribution of CDR3 in IGH genes. 

Here we demonstrate a new method for identifying clonally related sequences in large sets of rearranged IGH sequences, based on analysis of the highly variable CDR3 region of the VH domain. Sequences are partitioned using iHMMune-align [8] then clustered based on CDR3 similarity and common V and J genes. Clusters meeting an empirical quality criterion are then identified and extracted as sets of potentially clonally related sequences. This method is particularly well suited to the automated extraction of clonally related sequences sets from high throughput sequencing data.

## Results

A hierarchical agglomerative clustering method was implemented to group IG gene sequences on the basis of CDR3 sequence similarity and IGHV and IGHJ usage, with clusters below an empirically selected threshold classified as clonally related. The resulting software program can be downloaded from http://www.cse.unsw.edu.au/~ihmmune/ClonalRelate/ClonalRelate.zip. It accepts as input a set of sequences partitioned by iHMMune-align (as a semi-colon separated text file) and outputs a comma-separated text file listing the sequences and their clonal set assignment, together with dendrograms showing the structure of the clonal sets, in XML format. Several methods were tested for calculating a pairwise distance reflecting clonal relationships that was suitable for clustering. The resulting algorithms were evaluated using a benchmark sequence set containing known clonally-related sequence sets. The best performing version of the algorithm provided a more accurate identification of clonal sets than review by a domain expert. 

### Benchmark sequence set

In order to evaluate the suitability of clustering for identifying clonally-related sequence sets in large sets of IG genes, a human IGH sequence dataset known to contain multiple clonally-related sets obtained by Sanger sequencing (PNG dataset, Genbank HM773966-HM775073)(Wang et al., unpublished results)  was selected as a benchmark set to evaluate methods for clonally-related set identification. The dataset was scrutinised by a domain expert and sequences recognised as containing sequencing errors were removed. The resulting benchmark dataset contained 1116 human IGH sequences. Sequences were partitioned using the hidden Markov model based immunoglobulin gene sequence partitioning tool, iHMMune-align. Partitioning results were then examined, and clonally related sequence sets were identified by an iterative process of visual inspection by domain experts and automated clustering using the methods developed in this study. The final benchmark data set contained 182 sequences grouped into 66 clusters of clonally related sequences (Table 1).	 

**Table 1:** PNG benchmark dataset

Number of Clusters	Numbers of Sequences in a Cluster
1	16
1	7
1	6
2	5
3	4
16	3
42	2

Two additional sets of clonally related sequence, PW99 and PW57, derived from tonsillar IgD class-switched B cells, have been previously described [15]. These sets contained 57 and 99 unique sequences respectively with each set, known to be derived from the same V-D-J rearrangements. These two sets were used to calculate the threshold used to label clusters as clonally related.

### Distance measure selection

The major difference between hierarchical clustering algorithms is the measure of similarity between each pair of clusters and the underlying modelling of the clusters. We experimentally evaluated the performance of different distance measures to generate a hierarchical representation of the clonal relatedness of a set of sequences.

Measuring the similarity of DNA sequences can be considered as a problem of comparing two strings. The Edit distance also known as the Levenshtein distance [16], can be considered as a classic measure of the similarity of two strings. However the un-normalised Levenshtein distance (LD) can yield biased results when comparing sequences of different lengths. The post-normalized edit distance (PNED) attenuates this effect, but it tends to yield smaller values for comparisons between similar-length strings.  The normalized edit distance (NED) was tested in other fields (for example pattern recognition) and can be considered as a similarity measure that is really independent of the length of the comparison [17-19].  

Distance matrices were generated by pairwise comparison of CDR3s from sequences in the PNG benchmark dataset using un-normalised, post-normalised and normalised Levenshtein distances. The benchmark dataset sequences were then clustered based on these distance matrices and the resulting clusters were compared to the benchmark “expert” clustering. Comparisons were performed using a range of gap penalties (0 to 8) and the best results were obtained in all cases with a fixed gap penalty of 3 per gap (independent of gap length). This value was subsequently adopted for all other tests. Table 2 demonstrates that the Normalized Edit Distance provided a better clustering of clonally related sequence sets than the other two distance measures. 

**Table 2:** Comparison of distance measures for clustering immunoglobulin gene variable sequences

Clustering method	Number of clusters below the threshold	Number of sequences in clusters below threshold	Number of clusters different from benchmark set	Number of incorrectly assigned sequences	Correctly clustered sequences (%)
(a) Expert inspection	67	184	4	16	95.1
(b) LD	117	364	71	182	50.0
(c) PNED	93	258	36	76	70.5
(d) NED	78	211	15	29	85.9
(e) NED_VJ	70	190	4	8	95.8

Sequences in the benchmark PNG dataset were clustered using the following 4 methods (a) Expert inspection carried out by visual inspection of the partitioned gene segments without automated clustering. (b) LD: automated clustering based on pairwise Levenshtein Distance between CDR3 sequences. (c) PNED: automated clustering based on post-normalized edit distance. The Levenshtein Distances of each sequence pair is normalize by square root of the length of longer sequence in comparison. (d) NED: automated clustering based on the Normalized Edit Distance. (e) NED_VJ: automated clustering based on the Normalized Edit Distance, incorporating germline gene identity. Gap penalties of 3 were applied to each automated method. The resulting clusterings were evaluated relative to the “benchmark” clustering obtained by combination of automated clustering and visual inspection.

### Incorporating germline gene identity into the distance metric

The CDR3 is the most variable region of the immunoglobulin V domain, and was therefore selected as the main criterion for clonal relatedness. However clonally related sequences share the same component germline genes and therefore including this information into the comparison should improve its accuracy. IGHD germline gene identity was not used as a criterion as this gene is difficult to identify accurately [20] and it is completely contained within the CDR3. For IGHV and IGHJ, germline gene assignments obtained from iHMMune-align were compared for each pair of sequences. Penalties for mismatches at the level of the allele, gene and subgroup were used to modify the CDR3 edit distance as detailed in Methods. Several combinations of penalties were trialled and the best performing scoring scheme (5 for mismatching family, 3 for mismatching gene, 1 for mismatching allele) was selected. The resulting distance metric (NED_VJ) improved the accuracy of clonally-related set identification in the benchmark set relative to the unmodified distance (NED) (Table 2). 

### Distance threshold estimation

An agglomerative hierarchical clustering algorithm was used to group sequences on the basis of their pairwise distance and to construct a dendrogram. An average linkage scheme [21] was implemented to calculate the mean distance between each elements of each cluster. 

**Figure 1:**
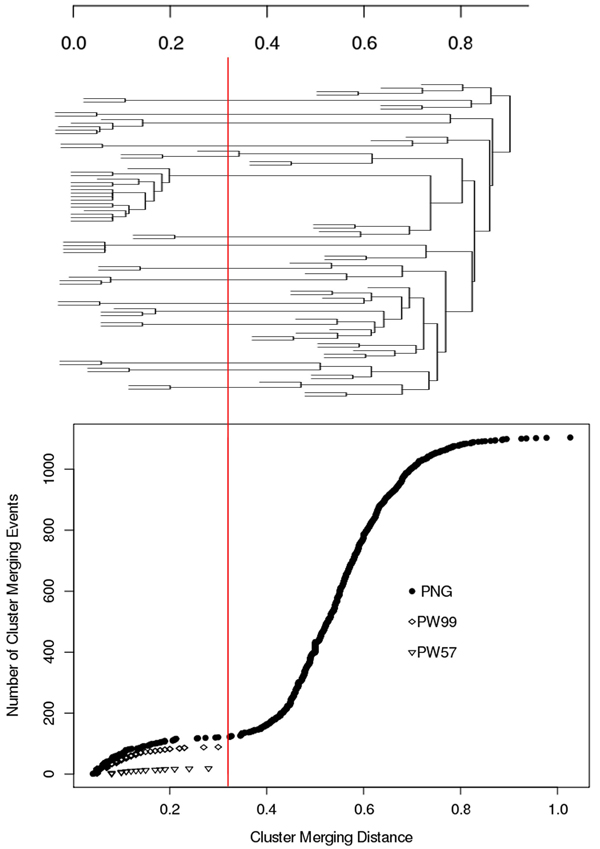
**Maximum distance (NED_VJ) within a cluster versus number of clusters processed for the PNG, PW99 and PW57 datasets.**  The red line corresponds to the distance threshold below which sequences are considered to be clonally-related.

In order to estimate the threshold distance below which a cluster corresponds to a clonally related sequence set, an evaluation graph showing the cumulative merging of sequences into clusters as the threshold distance between leaves is increased was plotted. This graph was constructed for the PNG, PW99 and PW57 datasets, using NED_VJ as similarity measure. Figure 1 shows this graph (derived from the whole dataset) juxtaposed with a sample dendrogram (consisting of 90 random selected sequences from PNG dataset) to clarify the merging process used in the evaluation graph. The PNG graph shows that after an initial rapid merging of clonally-related sequences into clusters, the plot flattens out as there are fewer similar sequences to merge into clusters. Once the merging distance increases to a level where the program starts clustering clonally-unrelated sequences the slope of the curve increases sharply. A cutoff distance of 0.32 (red line) allows good discrimination between clonally-related and unrelated sequences in the PNG set as well as correct clustering of all the sequences in the PW99 and PW57 sets. 

### Algorithm performance

The performance of the algorithm can be evaluated by evaluating the performance of the edit distance calculation algorithm and that of the agglomerative hierarchical clustering algorithm.

	Given two gene sequences X and Y of lengths *m* and *n*, and *n ≤ m.* Let LD(X, Y) be the Levenshtein Distance between X and Y, and NED(X,Y) be the normalized edit distance between X and Y. Using dynamic programming, the computation of  LD(X,Y) can be finished in *O(mn*) time and memory space The actual space requirements can be reduced to *O(n)* in implementation [17].  However, calculation of NED requires higher time and space complexity. According to Marzal and Vidal [17] NED requires computing NED(X,Y) in *O(m·n^2^)* time and an array of *(m+1) ·(n+1) ·(m+n)* memory locations. 

	The time and space complexities of calculating a distance matrix within a gene sequence dataset greatly depends on the size of the dataset *S*.  Time and space complexity will increase by *S*^2^.

	Agglomerative hierarchical clustering using average-link clustering merges in each iteration the pair of clusters with the highest cohesion. This algorithm does not scale well: the time complexity is at least *O(n^2^)*, where n is the number of total objects. 

	However CDR3 sequences are short sequences with lengths usually no more than 80 nucleotides. Therefore the computation of NED for each pair of sequences can be managed in a reasonably short time. The main limitation in the performance of the program is due to the large number of sequences being analysed. In our experiments with high-throughput data obtained by 454 sequencing [22], the largest datasets always comprised less than 5000 non-redundant sequences per individual. We have tested the performance of our program on the PNG dataset (1116 sequences) and on a dataset containing 4152 sequences, using a personal laptop computer with fairly standard specifications (Intel Core 2 Duo 2.53GHz, with 4GB RAM, Ubuntu Linux 8.04). The program was analysed the PNG dataset in 85.4 seconds and the large dataset in 1773 seconds (29.55 minutes). This level of performance is acceptable when considering the time required for generating and partitioning the sequence sets. 

## Discussion

B cell diversification is an integral part of the normal immune response and includes processes of somatic hypermutation (SHM) and isotype class switching within the germinal centres of the lymph nodes [20]. Understanding the origin and clonal evolution of B cells is critical to the overall understanding of the immune response in disease. A number of studies [3-5] have demonstrated the usefulness of analysing clonally-related immunoglobulin gene sequence sets in this context.

We designed a tool for identifying clonally related sets as a component in a pipeline for processing high-throughput rearranged immunoglobulin gene sequence data. This tool requires as input a set of sequences partitioned into their likely component germline gene using iHMMune-align, and labels these sequences with information about their likely clonally-related sequence cluster membership.  iHMMune-align is used to obtain the identity of the IGHV and IGHJ genes and was selected as it is a component of our analysis pipeline and the only currently available utility suitable for scriptable high-throughput analysis. However there is no reason why the clustering program cannot be modified to make use of other partitioning programs such as IMGT/V-QUEST or SoDA if these are available in high-throughput versions.

Standard phylogeny inference methods are not suitable for exploring clonal relationships within an immunoglobulin gene sequence dataset as antibodies diversify through processes that differ substantially from those of long time scale evolutionary events. However, string comparison methods that are widely used in sequence analysis are still applicable: sequence insertions, deletions and substitutions can be thought of as editing operations that transform one string into another. Pairwise distances between sequences can be calculated from string edit distances. Clonally related sequences are similar in length and have many common mutations, and this is reflected in their pairwise edit distances. A dendrogram representing the hierarchical structure of the relationship between sequences can then be constructed. 

The major differences between different hierarchical clustering algorithms are the measure of similarity between each pair of clusters and the underlying modelling of the clusters. Distance measures which can accurately represent relationships between sequences result in improved accuracy in the identification of clonally related sets. The Levenshtein distance is the most commonly used metric for measuring the dissimilarity between strings. However, it is not very suitable for strings of different lengths since it lacks normalization for appropriately weighting edit operations relative to the length of the strings being compared. It should be clear that one difference between two short strings is more critical than one difference between two longer strings.

There are two well-known approaches for normalizing the Levenshtein distance: one based on the editing path lengths (normalized edit distance, NED), and the other on the string lengths (post normalized edit distance, PNED). Post normalized edit distance (PNED) normalizes the editing transformation by the length of one of the strings, while NED normalizes the editing transformation by the alignment path length. Marzal and Vidal [17] demonstrated the improved accuracy of NED relative to PNED when comparing string patterns. Our results also demonstrated that NED resulted in improved accuracy when clustering clonally-related immunoglobulin gene sequences relative to PNED and to the un-normalized Levenshtein distance. 

The clustering accuracy was further improved by incorporating information about common usage of V and J genes between sequences, in addition to the CDR3 similarity. However CDR3 similarity remains the main criterion, as it is less sensitive to potential errors in germline gene identification. The resulting NED_VJ distance produced a clustering of clonally-related sequences in the benchmark set that, when examined by domain experts, was found to improve on their initial classification by visual inspection of the iHMMune-align outputs.  This demonstrates the value of our approach not only in automating the painstaking task of identifying clonally-related sequence sets by visual inspection but also in improving the accuracy of that identification.

Various combinations of values for the gap, V mismatch and J mismatch penalty parameters were tested using the PNG dataset in order to select the best performing values. The PNG dataset covers a wide range of germline gene usage and mutation level and is very likely to be representative of most heavy chain gene sets. The resulting values are therefore likely to be suitable for all immunoglobulin heavy chain gene sets. However the user has the option of modifying these values if deemed necessary.

One issue with clonally-related sequence identification is the presence of sets of sequences which appear clonally-related but are only similar as a result of chance matching. This is particularly an issue with sequences containing very short IGHD genes where CDR3 similarity can more readily occur by chance and is less likely to reflect a common clonal origin. This issue was addressed by selecting a conservative distance threshold below which a cluster is considered to be a clonally-related sequence set, although this may decrease the sensitivity of our method. Some improvements may be obtained by taking into account IGHD gene identity and number of shared mutations, when these can be determined with confidence [20].

Another source of error in the classification of the benchmark dataset was the presence of chimeric sequences in the dataset. These chimeric sequences are formed from recombination of two sequences and may have identical CDR3 sequences, but different V genes. Our method relies primarily on CDR3 differences for identifying clonally related sets, and therefore cannot avoid erroneous clustering of chimeric sequences. This problem was minimised by inclusion of IGHV identity in distance calculations.

## Conclusions

The identification of clonally related immunoglobulin gene sequences is usually performed by visual inspection of N1, IGHD and N2 regions from sequences previously identified as having the same IGHV and IGHJ regions, This visual inspection method is unsuitable for analysing high throughput sequencing data obtained using newly developed DNA sequencing technologies. To our knowledge, the hierarchical clustering based method presented here is the first automated method for clonally related immunoglobulin heavy chain gene identification. It identified clonally-related sequences with more accuracy than visual inspection alone, and successfully identified the known PW57 and PW99 clonally related sets when they were mixed into a much larger set of immunoglobulin gene sequences.

Our current method uses the most highly variable region in immunoglobulin gene, CDR3, as a key indicator of clonal relatedness and supplements it with information about shared V and J gene usage. Best results in terms of both speed and accuracy were obtained when pre-inspecting the input datasets to filter out sequencing errors and especially chimeric sequences. However, in the absence of an automated method for detecting such error sequences, our method can still be applied to unfiltered data sets as visual detection of chimeric sequences is easier on a dataset that has been processed to identify clonally-related sequences. In such a dataset, chimeric sequences typically show a pattern of IGHV mutations that differ significantly from the other sequences in their cluster and therefore stand out in the results.  

Clustering-based identification of clonal relatedness in immunoglobulin heavy chain genes improves our ability to study the clonal expansion in B cells, and provides a new tool for understanding the immune response in a large number of clinically important conditions including autoimmune diseases, infectious diseases and cancer

## Methods

### Immunoglobulin sequences sets

The PNG immunoglobulin sequence set was sampled from blood from a cohort of Papua New Guinea individuals. Sequences were visually inspected to filter out sequencing errors before running the test. The two other benchmark sets of clonally related sequences, PW99 and PW57, were derived from tonsillar IgD class-switched B cells [15], and consist of 57 and 99 unique sequences known to be derived from the same V-D-J rearrangements. 

### Construction of CDR3 starting and ending length libraries

The human germline IGHV and IGHJ repertoires were the same repertoires used by the current version of iHMMune-align. They include sequences obtained from IMGT [22,23,25] modified on the basis of a recent re-evaluation of the germline IGHV repertoire [22,26].

The CDR3 was defined as per the IMGT unique numbering [27], from position 105 to position 117 (codon or amino acid). This corresponds to the DNA regions between the IGHV region 2^nd^-CYS 104and the IGHJ region J-TRP 118. 

The IGHV germline repertoire was processed through IMGT/V-QUEST [6] to calculate, for each IGHV germline gene in the repertoire, the distance between the CDR3 start nucleotide and the end of the IGHV germline gene, which was stored in a CDR3 starting length library.

The CDR3 ending length library storing the distances between the start of each IGHJ germline gene and the end of CDR3 (defined as the first TGG (Trp) codon) was constructed in the same way. 

### Partitioning of immunoglobulin sequences

Rearranged V-D-J sequences were aligned to the reported germline repertoire using the iHMMune-align program, an alignment tool based on a hidden Markov model of the rearranged variable domain [8]. Sequences were partitioned into V, N1, D, N2 and J segments, and V/D/J segments were assigned to the most appropriate germline gene/allele. 

### Extraction of immunoglobulin CDR3 sequences

For each iHMMune-align-partitioned sequence, the CDR3 was extracted by concatenating the end of the IGHV region (as defined by the CDR3 starting length library), the N1, IGHD, N2 regions and the beginning of the IGHJ region (as defined by the CDR3 ending length library). 

### Calculation and normalisation of edit distance between CDR3 sequences

Levenshtein distance (LD), post-normalized edit distance (PNED) and normalized edit distance (NED) were calculated from pairs of CDR3 sequences using the described algorithms [16-18], implemented in a Java program.

### Calculation of NED-VJ distance

In the iHMMune-align results, V regions are identified by subgroup, gene and allele [28] (e.*g.* for IGHV1-2*01, subgroup is IGHV1, gene is IGHV1-2 and allele is IGHV1-2*01). IGHJ germline genes are not classified into families and are therefore labelled only with gene and allele numbers. 

The NED_VJ distance was calculated as:

Where LD is the un-normalised Levenshtein distance, *S_V_* is the mismatch penalty for IGHV germline gene (0 when the two sequences match the same IGHV gene, 1 when only the allele differs, 3 when the gene differs, and 5 for different subgroups), *S_J_* is the mismatch penalty for IGHJ gene (0 when the two sequences match the same IGHJ germline gene, 1 when they differ only in allele assignment and 3 when they match different genes). *L* is the CDR3 alignment length (edit path length).

### Hierarchical clustering

Agglomerative hierarchical clustering was performed using the mean distance between elements of each cluster (average linkage) to build a dendrogram for the sequence set. The algorithm was implemented in Java. The resulting dendrogram was stored as an XML tree file.

### Extraction of clonally related sequence sets

The distance threshold defining a clonally-related sequence cluster was determined by analysis of the overall agglomerative clustering pattern in the PNG dataset and using two sets of sequences known to be clonally-related (PW57 and PW99). Sub-clusters below this threshold were extracted and stored into XML sub-tree files. Clonally-related sequence sets were also output as a comma-delimited text file for import into a spreadsheet for further analysis.

## Competing interests

The authors declare no competing interests.

## Authors' contributions

ZC developed, implemented and tested the algorithm under the supervision of BG. YW and AC provided the benchmark datasets and expert evaluation of the method as well as feedback on its performance. All authors reviewed and agree with the final version of the manuscript.
